# Supplementary material for: No Evidence of the Vertical Transmission of Non-Virulent Infectious Salmon Anaemia Virus (ISAV-HPR0) in Farmed Atlantic Salmon
Source: Viruses. 2021 Dec 3;13(12):2428. doi: 10.3390/v13122428 (PMC8708482; doi:10.3390/v13122428)
Supplement: Supplementary file 1 [file viruses-13-02428-s001.zip › viruses-1422773-supplementary.pdf]

Table S1: Overview of the 235 ISAV-HPR0 variants included in the phylogenetic analysis in the present study including virus ID, year of collection, farm ID, subgroup by sequencing, subgroup by the G2- and G4-specific real-time RT-qPCR, GenBank accession numbers and published by.

Country/virus ID/year of detection	Year	Production stage (farm ID)	Subgroup by Sanger sequencing (SS) and/or Illumina next generation sequencing (NGS))	Subgroup by G2 and/or G4 Specific real-time RT-PCR (G2 Ct-values / G4 Ct-values)	GenBank Accession number	Source
Brood Fish						
NO/H97/04	2004	Brood fish (N/A)	G3 (SS)	N/A	DQ108604	Nylund et al. 2007, 2019
NO/H97b/04	2004	Brood fish (N/A)	G3 (SS)	N/A	MH397901	Nylund et al. 2019
NO/H97c/04	2004	Brood fish (N/A)	G3 (SS)	N/A	MH397902	Nylund et al. 2019
NO/H97d/04	2004	Brood fish (N/A)	G3 (SS)	N/A	MH397903	Nylund et al. 2019
NO/H97e/04	2004	Brood fish (N/A)	G3 (SS)	N/A	MH397904	Nylund et al. 2019
NO/MR102/05	2005	Brood fish (N/A)	G1 (SS)	N/A	DQ108605	Nylund *et al*. 2007, 2019
NO/MR102b/05	2005	Brood fish (N/A)	G1 (SS)	N/A	MH397906	Nylund et al. 2019
NO/MR102c/05	2005	Brood fish (N/A)	G1 (SS)	N/A	MH397907	Nylund et al. 2019
NO/MR102d/05	2005	Brood fish (N/A)	G1 (SS)	N/A	MH397908	Nylund et al. 2019
NO/MR102e/05	2005	Brood fish (N/A)	G1 (SS)	N/A	MH397909	Nylund et al. 2019
NO/MR104/05	2005	Brood fish (N/A)	G3 (SS)	N/A	DQ108607	Nylund *et al*. 2007, 2019
NO/SC1/08A	2008	Brood fish (N/A)	G3 (SS)	N/A	HE800147.1	Lyngstad *et al*. 2012
NO/SC1/08B	2008	Brood fish (N/A)	G3 (SS)	N/A	HE800148.1	Lyngstad *et al*. 2012
FO/509-1/08	2008	Brood fish I (BI)	G2c / G4a (SS)	G2 (27.5) / G4 (26.8)	OK349425	Present study
FO/509-2/08	2008	Brood fish I (BI)	G2c / G4a (SS)	G2 (25.8) / G4 (24.8)	OK349425	Present study
FO/509-3/08	2008	Brood fish I (BI)	G2c / G4a (SS)	G2 (25.6) / G4 (25.8)	OK349426	Present study
FO/510-1/08	2008	Brood fish I (BI)	G2c / G4 a(SS)	G2 (24.6) / G4 (23.9)	OK349427	Present study
FO/510-2/08	2008	Brood fish I (BI)	G2c / G4a (SS)	G2 (27.4) / G4 (26.9)	OK349428	Present study
FO/510-3a/08	2008	Brood fish I (BI)	G2c / G4a (SS + NGS)	G2 (22.6) / G4 (23.2)	OK349429	Present study
FO/510-3b/08	2008	Brood fish I (BI)	G2c / G4a (SS + NGS)	G2 (22.6) / G4 (23.2)	OK349430	Present study
FO/510-4/08	2008	Brood fish I (BI)	G2c / G4a (SS)	G2 (25.4) / G4 (24.8)	OK349431	Present study
FO/515-1/08	2008	Brood fish I (BI)	G2c / G4a (SS)	G2 (25.5) / G4 (22.9)	OK349432	Present study
FO/515-2/08	2008	Brood fish I (BI)	G2c / G4a (SS)	G2 (26.3) / G4 (22.8)	OK349433	Present study
FO/515-3/08	2008	Brood fish I (BI)	G2c / G4a (SS)	G2 (19.0) / G4 (17.8)	OK349434	Present study
FO/515-4/08	2008	Brood fish I (BI)	G2c / G4a (SS)	G2 (19.6) / G4 (17.8)	OK349435	Present study
FO/531-1/08	2008	Brood fish I (BI)	G2c / G4a (SS)	G2 (21.3) / G4 (17.5)	OK349436	Present study
FO/531-2/08	2008	Brood fish I (BI)	G4a / G4a (SS)	G2 (16.5) / G4 (14.6)	OK349437	Present study
FO/574-1/10	2010	Brood fish I (BI)	G2d (SS)	G2 (26.8) / G4 (neg.)	OK349438	Present study
FO/574-2/10	2010	Brood fish I (BI)	G2d (SS)	G2 (27.4) / G4 (neg.)	OK349439	Present study
FO/574-3/10	2010	Brood fish I (BI)	G2d (SS)	G2 (26.6) / G4 (neg.)	OK349440	Present study
FO/574-4/10	2010	Brood fish I (BI)	G2d (SS)	G2 (27.2) / G4 (neg.)	OK349441	Present study
FO/578-1/10	2010	Brood fish I (BI)	G2d (SS)	G2 (26.6) / G4 (neg.)	OK349442	Present study
FO/578-2/10	2010	Brood fish I (BI)	G2d (SS)	G2 (27.9) / G4 (neg.)	OK349443	Present study
FO/578-3/10	2010	Brood fish I (BI)	G2d (SS)	G2 (27.6) / G4 (neg.)	OK349444	Present study

FO/578-4/10	2010	Brood fish I (BI)	G2d (SS)	G2 (26.4) / G4 (neg.)	OK349445	Present study
FO/686-1/10	2010	Brood fish I (BI)	G2d (NGS)	G2 (16.7) / G4 (neg.)	OK349446	Present study
FO/771-1/10	2010	Brood fish I (BI)	G2d (SS)	G2 (15.8) / G4 (neg.)	OK349447	Present study
FO/771-2/10	2010	Brood fish I (BI)	G2d (NGS)	G2 (18.1) / G4 (neg.)	OK349448	Present study
FO/771-3/10	2010	Brood fish I (BI)	G2d (SS)	G2 (18.5) / G4 (neg.)	OK349449	Present study
FO/67-1/12	2012	Brood fish I (BI)	G2d (SS)	G2 (22.0) / G4 (neg.)	OK349450	Present study
FO/67-2/12	2012	Brood fish I (BI)	G2d (SS)	G2 (24.0) / G4 (neg.)	OK349451	Present study
FO/67-3/12	2012	Brood fish I (BI)	G2d (SS)	G2 (21.8) / G4 (neg.)	OK349452	Present study
FO/67-4/12	2012	Brood fish I (BI)	G2d (SS)	G2 (23.3) / G4 (neg.)	OK349453	Present study
FO/85-1/12	2012	Brood fish I (BI)	G2d (SS)	G2 (23.4) / G4 (neg.)	OK349454	Present study
FO/85-2/12	2012	Brood fish I (BI)	G2d (SS)	G2 (24.8) / G4 (neg.)	OK349455	Present study
IS/530-1/09	2009	Brood fish II (BII)	G2b (SS)	N/A	OK349456	Present study
IS/532-1/09	2009	Brood fish II (BII)	G2b (SS)	N/A	OK349457	Present study
IS/532-2/09	2009	Brood fish II (BII)	G2b (SS)	N/A	OK349458	Present study
IS/533-1/09	2009	Brood fish II (BII)	G2b (SS)	N/A	OK349459	Present study
IS/569-1/09	2009	Brood fish II (BII)	G2b (SS)	N/A	OK349460	Present study
IS/571-1/09	2009	Brood fish II (BII)	G2b (SS)	N/A	OK349461	Present study
IS/571-2/09	2009	Brood fish II (BII)	G2b (SS)	N/A	OK349462	Present study
IS/572-1/09	2009	Brood fish II (BII)	G2b (SS)	N/A	OK349463	Present study
IS/574-1/09	2009	Brood fish II (BII)	G2b (SS)	N/A	OK349464	Present study
IS/574-2/09	2009	Brood fish II (BII)	G2b (SS)	N/A	OK349465	Present study
IS/576-1/09	2009	Brood fish III (BIII)	G2a (SS)	N/A	OK349466	Present study
IS/576-2/09	2009	Brood fish III (BIII)	G2a (SS)	N/A	OK349467	Present study
IS/577-1/09	2009	Brood fish III (BIII)	G2a (SS + NGS)	N/A	OK349468	Present study
IS/577-2/09	2009	Brood fish III (BIII)	G2a (SS)	N/A	OK349469	Present study
IS/577-3/09	2009	Brood fish III (BIII)	G2a (SS)	N/A	OK349470	Present study
IS/669-1/09	2009	Brood fish III (BIII)	G2a (SS)	N/A	OK349471	Present study
IS/578-1/09	2009	Brood fish II (BIII)	G2a (SS)	N/A	OK349472	Present study
Smolt fish						
FO/472-1/09	2009	Smolt farm I (SI)	G4 (SS)	G2 (neg.) / G4 (26.8)	OL614013	Present study
FO/472-2/09	2009	Smolt farm I (SI)	G4 (SS)	G2 (neg.) / G4 (28.1)	OL614014	Present study
FO/472-3/09	2009	Smolt farm I (SI)	G4 (SS)	G2 (neg.) / G4 (29.4)	OL614015	Present study
FO/472-4/09	2009	Smolt farm I (SI)	G4 (SS)	G2 (neg.) / G4 (28.1)	OL614016	Present study
FO/159-1/10	2010	Smolt farm I (SI)	G4 (SS)	G2 (neg.) / G4 (27.4)	OL614017	Present study
FO/83-1a/10	2010	Smolt farm II (SII)	G4 (SS)	G2 (neg.) / G4 (21.9)	OL614018	Present study
FO/83-1b/10	2010	Smolt farm II (SII)	G4 (SS)	G2 (neg.) / G4 (21.9)	OL614019	Present study
FO/83-2a/10	2010	Smolt farm II (SII)	G4 (SS)	G2 (neg.) / G4 (22.8)	OL614020	Present study
FO/83-2b/10	2010	Smolt farm II (SII)	G4 (SS)	G2 (neg.) / G4 (22.8)	OL614021	Present study
FO/83-3a/10	2010	Smolt farm II (SII)	G4 (SS + NGS)	G2 (neg.) / G4 (20.7)	OL614022	Present study
FO/83-3b/10	2010	Smolt farm II (SII)	G4 (SS + NGS)	G2 (neg.) / G4 (20.7)	OL614023	Present study

FO/83-4a/10	2010	Smolt farm II (SII)	G4 (SS)	G2 (neg.) / G4 (18.8)	OL614024	Present study
FO/83-4b/10	2010	Smolt farm II (SII)	G4 (SS)	G2 (neg.) / G4 (18.8)	OL614025	Present study
FO/648-1/10	2010	Smolt farm II (SII)	G4 (NGS)	G2 (neg.) / G4 (21.8)	OL614026	Present study
FO/648-2/10	2010	Smolt farm II (SII)	G4 (SS)	G2 (neg.) / G4 (24.5)	OL614027	Present study
FO/648-3/10	2010	Smolt farm II (SII)	G4 (SS + NGS)	G2 (neg.) / G4 (20.7)	OL614028	Present study
FO/007-1/12	2012	Smolt farm II (SII)	G4c (SS)	G2 (neg.) / G4 (24.2)	KX823921	Christiansen *et al.* 2017
FO/007-2/12	2012	Smolt farm II (SII)	G4c (SS + NGS)	G2 (neg.) / G4 (23.7)	OL614029	Present study
FO/007-3/12	2012	Smolt farm II (SII)	G4c (SS)	G2 (neg.) / G4 (19.4)	OL614030	Present study
FO/007-4/12	2012	Smolt farm II (SII)	G4c (SS + NGS)	G2 (neg.) / G4 (21.1)	OL614031	Present study
FO/007-5/12	2012	Smolt farm II (SII)	G4c (SS + NGS)	G2 (neg.) / G4 (19.5)	OL614032	Present study
FO/586-1/13	2013	Smolt farm II (SII)	G4c (SS)	G2 (neg.) / G4 (29.4)	OL614033	Present study
FO/586-2/13	2013	Smolt farm II (SII)	G4c (SS + NGS)	G2 (neg.) / G4 (30.4)	OL614034	Present study
FO/1300-1/14	2014	Smolt farm II (SII)	G4c (NGS)	G2 (neg.) / G4 (30.6)	OL614035	Present study
FO/1300-2/14	2014	Smolt farm II (SII)	G4c (NGS)	G2 (neg.) / G4 (26.0)	OL614036	Present study
FO/401-1a/14	2014	Smolt farm IV (SIV)	G4 (NGS)	G2 (neg.) / G4 (26.3)	OL614037	Present study
FO/401-1b/14	2014	Smolt farm IV (SIV)	G4a (NGS)	G2 (neg.) / G4 (26.3)	OL614038	Present study
FO/1256-1a/14	2014	Smolt farm IV (SIV)	G4 (SS + NGS)	G2 (neg.) / G4 (21.1)	OL614039	Present study
FO/1256-1b/14	2014	Smolt farm IV (SIV)	G4a (SS + NGS)	G2 (neg.) / G4 (21.1)	OL614040	Present study
FO/1256-2a/14	2014	Smolt farm IV (SIV)	G4 (SS)	G2 (neg.) / G4 (26.3)	OL614041	Present study
FO/1256-2b/14	2014	Smolt farm IV (SIV)	G4a (SS)	G2 (neg.) / G4 (26.3)	OL614042	Present study
FO/1256-3a/14	2014	Smolt farm IV (SIV)	G4 (SS)	G2 (neg.) / G4 (24.5)	OL614043	Present study
FO/1256-3b/14	2014	Smolt farm IV (SIV)	G4a (SS)	G2 (neg.) / G4 (24.5)	OL614044	Present study
FO/1256-4a/14	2014	Smolt farm IV (SIV)	G4 (SS)	G2 (neg.) / G4 (25.7)	OL614045	Present study
FO/1256-4b/14	2014	Smolt farm IV (SIV)	G4a (SS)	G2 (neg.) / G4 (25.7)	OL614046	Present study
FO/1256-5/14	2014	Smolt farm IV (SIV)	G4 (SS)	G2 (neg.) / G4 (28.8)	OL614047	Present study
FO/1256-6/14	2014	Smolt farm IV (SIV)	G4 (SS)	G2 (neg.) / G4 (29.7)	OL614048	Present study
FO/1256-7/14	2014	Smolt farm IV (SIV)	G4 (SS)	G2 (neg.) / G4 (26.7)	OL614049	Present study
FO/1256-8/14	2014	Smolt farm IV (SIV)	G4 (SS)	G2 (neg.) / G4 (27.8)	OL614050	Present study
FO/156-1/08	2008	Smolt farm V (SV)	G2c (SS)	G2 (28.9) / G4 (neg.)	OL614051	Present study
FO/156-2/08	2008	Smolt farm V (SV)	G2c (SS)	G2 (31.7) / G4 (neg.)	OL614052	Present study
FO/156-3/08	2008	Smolt farm V (SV)	G4a (SS)	G2 (28.9) / G4 (26.8)	OL614053	Present study
FO/156-4/08	2008	Smolt farm V (SV)	G2c (SS)	G2 (30.0) / G4 (36.5)	OL614054	Present study
FO/82-1/10	2010	Smolt farm V (SV)	G4 (SS)	G2 (neg.) / G4 (25.7)	OL614055	Present study
FO/82-2/10	2010	Smolt farm V (SV)	G4 (SS)	G2 (neg.) / G4 (27.4)	OL614056	Present study
FO/160-1/10	2010	Smolt farm V (SV)	G4 (SS)	G2 (neg.) / G4 (25.8)	OL614057	Present study
FO/401-1/10	2010	Smolt farm V (SV)	G4 (SS)	G2 (neg.) / G4 (23.8)	OL614058	Present study
FO/401-2/10	2010	Smolt farm V (SV)	G4 (SS)	G2 (neg.) / G4 (27.4)	OL614059	Present study
FO/452-1/11	2011	Smolt farm V (SV)	G4 (SS)	G2 (neg.) / G4 (27.8)	KX823927	Christiansen *et al.* 2017
FO/452-2/11	2011	Smolt farm V (SV)	G4 (SS)	G2 (neg.) / G4 (27.4)	OL614060	Present study
FO/710-1/11	2011	Smolt farm V (SV)	G4 (SS)	G2 (neg.) / G4 (26.0)	OL614061	Present study
FO/128-1/09	2009	Smolt farm VI (SVI)	G4 (SS)	G2 (neg.) / G4 (17.9)	OL614062	Present study

FO/128-2/09	2009	Smolt farm VI (SVI)	G4 (SS)	G2 (neg.) / G4 (16.9)	OL614063	Present study
**FO/455-1/11**	2011	Smolt farm VI (SVI)	G4 (SS + NGS)	G2 (neg.) / G4 (28.6)	KX823928	Christiansen *et al*. 2017
FO/455-2/11	2011	Smolt farm VI (SVI)	G4 (SS)	G2 (neg.) / G4 (25.2)	OL614064	Present study
FO/455-3/11	2011	Smolt farm VI (SVI)	G4 (SS)	G2 (neg.) / G4 (24.8)	OL614065	Present study
FO/455-4/11	2011	Smolt farm VI (SVI)	G4 (SS)	G2 (neg.) / G4 (23.7)	OL614066	Present study
FO/455-5/11	2011	Smolt farm VI (SVI)	G4 (SS)	G2 (neg.) / G4 (27.8)	OL614067	Present study
**FO/570-1/12**	2011	Smolt farm VI (SVI)	G4b (SS + NGS)	G2 (neg.) / G4 (21.0)	KX823929	Christiansen *et al*. 2017
FO/570-2/12	2012	Smolt farm VI (SVI)	G4b (SS)	G2 (neg.) / G4 (27.6)	OL614068	Present study
**FO/1192-1/14**	2014	Smolt farm VI (SVI)	G4b (SS)	G2 (neg.) / G4 (26.7)	OL614069	Present study
FO/1192-2/14	2014	Smolt farm VI (SVI)	G4b (SS)	G2 (neg.) / G4 (25.6)	OL614070	Present study
**FO/1192-3/14**	2014	Smolt farm VI (SVI)	G4b (SS)	G2 (neg.) / G4 (27.7)	OL614071	Present study
FO/1192-4/14	2014	Smolt farm VI (SVI)	G4b (SS)	G2 (neg.) / G4 (25.3)	OL614072	Present study
Marine fish						
FO/230-1/07	2007	Marine farm I (MI)	G2 (SS)	G2 (32.9) / G4 (33.5)	OL631651	Present study
FO/230-2/07	2007	Marine farm I (MI)	G4 (SS)	G2 (33.9) / G4 (27.5)	OL631652	Present study
FO/285-1/07	2007	Marine farm I (MI)	G2 (SS)	G2 (33.8) / G4 (neg.)	OL631653	Present study
FO/327-1/07	2007	Marine farm I (MI)	G2 (SS)	G2 (31.4) / G4 (33.6)	OL631654	Present study
FO/327-2/07	2007	Marine farm I (MI)	G2 (SS)	G2 (31.3) / G4 (36.4)	OL631655	Present study
FO/40-1/08	2008	Marine farm I (MI)	G4 (SS)	G2 (neg.) / G4 (33.3)	OL631656	Present study
FO/40-2/08	2008	Marine farm I (MI)	G4 (SS)	G2 (neg.) / G4 (32.8)	OL631657	Present study
FO/138-1/08	2008	Marine farm I (MI)	G4 (SS)	G2 (33.6) / G4 (27.6)	OL631658	Present study
FO/149-1/08	2008	Marine farm I (MI)	G4 (SS)	G2 (neg.) / G4 (28.7)	OL631659	Present study
FO/149-2/08	2008	Marine farm I (MI)	G4 (SS)	G2 (31.0) / G4 (19.9)	OL631660	Present study
FO/149-3/08	2008	Marine farm I (MI)	G4 (SS)	G2 (neg.) / G4 (30.7)	OL631661	Present study
FO/166-1/07	2007	Marine farm II (MII)	G2 (SS)	G2 (26.7) / G4 (neg.)	OL631662	Present study
FO/166-2/07	2007	Marine farm II (MII)	G2 (SS)	G2 (24.4) / G4 (26.4)	OL631663	Present study
FO/238-1/07	2007	Marine farm II (MII)	G2 (SS)	G2 (26.6) / G4 29.3)	OL631664	Present study
FO/489-1/09	2009	Marine farm II (MII)	G4 (SS)	G2 (neg.) / G4 (20.5)	OL631665	Present study
FO/489-2/09	2009	Marine farm II (MII)	G4 (SS)	G2 (neg.) / G4 (28.9)	OL631666	Present study
FO/596-1/09	2009	Marine farm II (MII)	G4 (SS)	G2 (neg.) / G4 (24.4)	OL631667	Present study
FO/596-2/09	2009	Marine farm II (MII)	G4 (SS)	G2 (neg.) / G4 (25.5)	OL631668	Present study
FO/596-3/09	2009	Marine farm II (MII)	G4 (SS)	G2 (neg.) / G4 (23.6)	OL631669	Present study
FO/178-1/14	2014	Marine farm III (MIII)	G4 (SS)	G2 (32.4) / G4 (26.7)	KX823935	Christiansen et al. 2017
FO/178-2/14	2014	Marine farm III (MIII)	G4 (SS)	G2 (32.7) / G4 (26.5)	OL631670	Present study
FO/178-3/14	2014	Marine farm III (MIII)	G4 (SS)	G2 (30.5) / G4 (24.0)	OL631671	Present study
FO/211-1/07	2007	Marine farm IV (MIV)	G2 (SS)	G2 (29.3) / G4 (neg.)	OL631672	Present study
FO/270-1/07	2007	Marine farm IV (MIV)	G2 (SS)	G2 (31.2) / G4 (neg.)	OL631673	Present study
FO/270-2/07	2007	Marine farm IV (MIV)	G2 (SS)	G2 (27.7) / G4 (29.6)	OL631674	Present study
FO/270-3/07	2007	Marine farm IV (MIV)	G2 (SS)	G2 (31.6) / G4 (36.3)	OL631675	Present study
FO/319-1/07	2007	Marine farm IV (MIV)	G2 (SS)	G2 (27.5) / G4 (29.0)	OL631676	Present study
FO/319-2/07	2007	Marine farm IV (MIV)	G2 (SS)	G2 (30.4) / G4 (33.7)	OL631677	Present study

FO/319-3/07	2007	Marine farm IV (MIV)	G2 (SS)	G2 (29.3) / G4 (32.6)	OL631678	Present study
FO/728-1/12	2012	Marine farm V (MV)	G4 (SS)	G2 (neg.) / G4 (28.9)	OL631679	Present study
FO/1123-1/13	2013	Marine farm V (MV)	G4 (SS)	G2 (neg.) / G4 (26.6)	OL631680	Present study
FO/315-1/07	2007	Marine farm VI (MVI)	G4 (SS)	G2 (neg.) / G4 (27.8)	HQ664998	Christiansen et al. 2011
FO/365-1/07	2007	Marine farm VI (MVI)	G2 (SS)	G2 (33.3) / G4 (neg.)	OL631681	Present study
FO/521-1/07	2007	Marine farm VI (MVI)	G2 (SS)	G2 (31.9) / G4 (neg.)	OL631682	Present study
FO/286-1/09	2009	Marine farm VI (MVI)	G2 (SS)	G2 (26.4) / G4 (neg.)	OL631683	Present study
FO/286-2/09	2009	Marine farm VI (MVI)	G4 (SS)	G2 (neg.) / G4 (28.8)	OL631684	Present study
FO/97-1/10	2010	Marine farm VI (MVI)	G2 (SS)	G2 (26.9) / G4 (24.5)	OL631685	Present study
FO/97-2/10	2010	Marine farm VI (MVI)	G4 (SS)	G2 (30.3) / G4 (25.3)	OL631686	Present study
FO/249-1/12	2012	Marine farm VI (MVI)	G4 (SS)	G2 (neg.) / G4 (31.7)	OL631687	Present study
FO/482-1/08	2008	Marine farm VII (MVII)	G2 (SS)	G2 (21.9) / G4 (27.7)	OL631688	Present study
FO/482-2/08	2008	Marine farm VII (MVII)	G2 (SS)	G2 (22.2) / G4 (31.0)	OL631689	Present study
FO/615-1/09	2009	Marine farm VII (MVII)	G4 (SS)	G2 (neg.) / G4 (25.3)	OL631690	Present study
FO/242-1/09	2009	Marine farm VIII (MVIII)	G4 (SS)	G2 (27.6) / G4 (19.6)	OL631691	Present study
FO/242-2/09	2009	Marine farm VIII (MVIII)	G4 (SS)	G2 (30.5) / G4 (21.7)	OL631692	Present study
FO/64-1/12	2012	Marine farm VIII (MVIII)	G4 (SS)	G2 (neg.) / G4 (30.6)	OL631693	Present study
FO/458-1/07	2007	Marine farm IX (MIX)	G2 (SS)	G2 (32.6) / G4 (neg.)	OL631694	Present study
FO/547-1/08	2008	Marine farm IX (MIX)	G2 (SS)	G2 (27.7) / G4 (neg.)	OL631695	Present study
FO/547-2/08	2008	Marine farm IX (MIX)	G2 (SS)	G2 (25.9) / G4 (neg.)	OL631696	Present study
FO/457-1/09	2009	Marine farm IX (MIX)	G4 (SS)	G2 (neg.) / G4 (20.0)	OL631697	Present study
FO/457-2/09	2009	Marine farm IX (MIX)	G4 (SS)	G2 (neg.) /G4 (22.4)	OL631698	Present study
FO/453-1/08	2008	Marine farm X (MX)	G4 (SS)	G2 (neg.) / G4 (29.2)	OL631699	Present study
FO/453-2/08	2008	Marine farm X (MX)	G4 (SS)	G2 (neg.) / G4 (27.8)	OL631700	Present study
FO/453-3/08	2008	Marine farm X (MX)	G4 (SS)	G2 (neg.) / G4 (24.6)	OL631701	Present study
FO/503-1/07	2007	Marine farm XI (MXI)	G2 (SS)	G2 (31.6) / G4 (32.4)	OL631702	Present study
FO/518-1/07	2007	Marine farm XI (MXI)	G2 (SS)	G2 (24.9) / G4 (26.3)	OL631703	Present study
FO/518-2/07	2007	Marine farm XI (MXI)	G2 (SS)	G2 (22.3) / G4 (22.0)	OL631704	Present study
FO/92-1/10	2010	Marine farm XI (MXI)	G4 (SS)	G2 (27.3) / G4 (23.6)	OL631705	Present study
FO/92-2/10	2010	Marine farm XI (MXI)	G4 (SS)	G2 (neg.) / G4 (24.5)	OL631706	Present study
FO/466/12	2012	Marine farm XI (MXI)	G4 (SS)	G2 (neg.) / G4 (28.6)	OL631707	Present study
FO/1407-1/14	2014	Marine farm XI (MXI)	G4 (SS)	G2 (31.6) / G4 (27.3)	OL631708	Present study
FO/452-1/08	2008	Marine farm XII (MXII)	G4 (SS)	G2 (36.5) / G2 (29.5)	OL631709	Present study
FO/452-2/08	2008	Marine farm XII (MXII)	G2 (SS)	G2 (28.5) / G4 (neg.)	OL631710	Present study
FO/551-1/10	2010	Marine farm XII (MXII)	G2 (SS)	G2 (27.8) / G4 (neg.)	OL631711	Present study
FO/551-2/10	2010	Marine farm XII (MXII)	G2 (SS)	G2 (29.5) / G4 (neg.)	OL631712	Present study
FO/862-1/12	2012	Marine farm XII (MXII)	G2 (SS)	G2 (28.9) / G4 (neg.)	OL631713	Present study
FO/862-2/12	2012	Marine farm XII (MXII)	G2 (SS)	G2 (26.9) / G4 (neg.)	OL631714	Present study
FO/862-3/12	2012	Marine farm XII (MXII)	G2 (SS)	G2 (23.8) / G4 (26.9)	OL631715	Present study
FO/1120-1/13	2013	Marine farm XII (MXII)	G4 (SS)	G2 (neg.) / G4 (24.4)	OL631716	Present study
FO/1120-2/13	2013	Marine farm XII (MXII)	G4 (SS)	G2 (neg.) / G4 (20.8)	OL631717	Present study

FO/1120-3/13	2013	Marine farm XII (MXII)	G4 (SS)	G2 (neg.) / G4 (28.5)	OL631718	Present study
FO/549-1/08	2008	Marine farm XIII (MXIII)	G2 (SS)	G2 (28.4) / G4 (neg.)	HQ664999	Christiansen et al. 2011
FO/55-1/08	2008	Marine farm XIII (MXIII)	G2 (SS)	G2 (24.3) / G4 (neg.)	OL631719	Present study
FO/55-2/08	2008	Marine farm XIII (MXIII)	G2 (SS)	G2 (26.7) / G4 (neg.)	OL631720	Present study
FO/592-1/08	2008	Marine farm XIV (MXIV)	G4 (SS)	G2 (28.3) / G4 (23.5)	OL631721	Present study
FO/592-2/08	2008	Marine farm XIV (MXIV)	G4 (SS)	G2 (35.4) / G4 (23.5)	OL631722	Present study
FO/592-3/08	2008	Marine farm XIV (MXIV)	G4 (SS)	G2 (28.7) / G4 (23.7)	OL631723	Present study
FO/59-1/11	2011	Marine farm XIV (MXIV)	G2 (SS)	G2 (24.6) / G4 (26.7)	OL631724	Present study
FO/143-1/14	2014	Marine farm XIV (MXIV)	G4 (SS)	G2 (32.5) / G4 (29.3)	OL631725	Present study
FO/143-2/14	2014	Marine farm XIV (MXIV)	G4 (SS)	G2 (33.5) / G4 (28.2)	KX823932	Christiansen et al. 2017
FO/214-1/07	2007	Marine farm XV (MXV)	G4 (SS)	N/A	HQ664995	Christiansen et al. 2011
FO/214-2/07	2007	Marine farm XV (MXV)	G4 (SS)	N/A	OL631726	Present study
FO/214-3/07	2007	Marine farm XV (MXV)	G2 (SS)	N/A	HQ664994	Christiansen et al. 2011
FO/214-4/07	2007	Marine farm XV (MXV)	G2 (SS)	N/A	OL631727	Present study
FO/214-5/07	2007	Marine farm XV (MXV)	G4 (SS)	N/A	OL631728	Present study
FO/384-1/08	2008	Marine farm XV (MXV)	G4 (SS)	G2 (27.2) / G4 (24.8)	OL631729	Present study
FO/384-2/08	2008	Marine farm XV (MXV)	G4 (SS)	G2 (28.8) / G4 (24.6)	OL631730	Present study
FO/330-1/14	2014	Marine farm XV (MXV)	G4 (SS)	G2 (neg.) / G4 (27.9)	OL631731	Present study
FO/330-2/14	2014	Marine farm XV (MXV)	G4 (SS)	G2 (neg.) / G4 (29.6)	OL631732	Present study
FO/371-1/07	2007	Marine farm XVI (MXVI)	G2 (SS)	G2 (35.3) / G4 (neg.)	OL631733	Present study
FO/371-2/07	2007	Marine farm XVI (MXVI)	G2 (SS)	G2 (32.7) / G4 (neg.)	OL631734	Present study
FO/506-1/07	2007	Marine farm XVI (MXVI)	G4 (SS)	G2 (29.9) / G4 (29.4)	OL631735	Present study
FO/506-2/07	2007	Marine farm XVI (MXVI)	G2 (SS)	G2 (25.5) / G4 (31.3)	OL631736	Present study
FO/504-1/06	2006	Marine farm XVII (MXVII)	G2 (SS)	N/A	HQ664992	Christiansen et al. 2011
FO/504-2/06	2006	Marine farm XVII (MXVII)	G2 (SS)	N/A	OL631737	Present study
FO/137-1/08	2008	Marine farm XVIII (MXVIII)	G4 (SS)	G2 (neg.) / G4 (29.3)	OL631738	Present study
FO/197-1/11	2011	Marine farm XVIII (MXVIII)	G4 (SS)	G2 (27.3) / G4 (23.6)	OL631739	Present study
FO/255-1/10	2010	Marine farm XIX (MXIX)	G2 (SS)	G2 (27.3) / G4 (28.8)	OL631740	Present study
FO/255-2/10	2010	Marine farm XIX (MXIX)	G2 (SS)	G2 (26.4) / G4 (28.5)	OL631741	Present study
FO/891-1/12	2012	Marine farm XIX (MXIX)	G4 (SS)	G2 (neg.) / G4 (29.6)	OL631742	Present study
FO/282-1/14	2014	Marine farm XIX (MXIX)	G4 (SS)	G2 (neg.) / G4 (27.6)	OL631743	Present study
FO/282-2/14	2014	Marine farm XIX (MXIX)	G4 (SS)	G2 (neg.) / G4 (29.4)	OL631744	Present study
FO/176-1/07	2007	Marine farm XX (MXX)	G4 (SS)	G2 (34.7) / G4 (29.8)	OL631745	Present study
FO/355-1/07	2007	Marine farm XX (MXX)	G4 (SS)	G2 (neg.) / G4 (29.5)	OL631746	Present study
FO/355-2/07	2007	Marine farm XX (MXX)	G2 (SS)	G2 (31.7) / G4 (neg.)	OL631747	Present study
FO/452-1/07	2007	Marine farm XX (MXX)	G2 (SS)	G2 (28.8) / G4 (36.3)	OL631748	Present study
FO/452-2/07	2007	Marine farm XX (MXX)	G4 (SS)	G2 (35.2) / G4 (28.6)	OL631749	Present study
FO/452-3/07	2007	Marine farm XX (MXX)	G2 (SS)	G2 (28.4) / G4 (32.8)	OL631750	Present study
FO/181-1/09	2009	Marine farm XX (MXX)	G2 (SS)	G2 (24.6) / G4 (26.6)	OL631751	Present study
FO/201-1/09	2009	Marine farm XX (MXX)	G4 (SS)	G2 (23.7) / G4 (21.7)	OL631752	Present study
FO/201-2/09	2009	Marine farm XX (MXX)	G2 (SS)	G2 (24.3) / G4 (28.4)	OL631753	Present study

FO/96-1/11	2011	Marine farm XX (MXX)	G4 (SS)	G2 (neg.) / G4 (28.7)	OL631754	Present study
FO/147-1/07	2007	Marine farm XXI (MXXI)	G2 (SS)	G2 (27.7) / G4 (32.3)	HQ664996	Christiansen et al. 2011
FO/147-2/07	2007	Marine farm XXI (MXXI)	G4 (SS)	G2 (35.4) / G4 (30.7)	HQ664997	Christiansen et al. 2011
FO/462-1/08	2008	Marine farm XXI (MXXI)	G4 (SS)	G2 (neg.) / G4 (30.6)	**OL631755**	Present study
FO/462-2/08	2008	Marine farm XXI (MXXI)	G4 (SS)	G2 (neg.) / G4 (28.4)	**OL631756**	Present study
FO/593-1/08	2008	Marine farm XXII (MXXII)	G4 (SS)	G2 (neg.) / G4 (29.0)	**OL631757**	Present study
FO/593-2/08	2008	Marine farm XXII (MXXII)	G4 (SS)	G2 (24.6) / G4 (21.3)	**OL631758**	Present study
FO/593-3/08	2008	Marine farm XXII (MXXII)	G4 (SS)	G2 (33.6) / G4 (23.0)	**OL631759**	Present study
FO/593-4/08	2008	Marine farm XXII (MXXII)	G4 (SS)	G2 (neg.) / G4 (25.9)	**OL631760**	Present study
FO/95-1/14	2014	Marine farm XXII (MXXII)	G4 (SS)	G2 (28.8) / G4 (22.4)	**OL631761**	Present study
FO/95-2/14	2014	Marine farm XXII (MXXII)	G4 (SS)	G2 (31.8) / G4 (27.4)	**OL631762**	Present study
FO/95-3a/14	2014	Marine farm XXII (MXXII)	G4 (SS)	G2 (27.3) / G4 (25.0)	KX823930	Christiansen et al. 2017
FO/95-3b/14	2014	Marine farm XXII (MXXII)	G2 (SS)	G2 (27.3) / G4 (25.0)	KX823931	Christiansen et al. 2017
FO/193-1/07	2007	Marine farm XXIII (MXXIII)	G2 (SS)	N/A	**OL631763**	Present study
FO/193-2/07	2007	Marine farm XXIII (MXXIII)	G2 (SS)	G2 (28.4) / G4 (36.4)	**OL631764**	Present study
FO/60-1/10	2010	Marine farm XXIII (MXXIII)	G4 (SS)	G2 (neg.) / G4 (25.0)	**OL631765**	Present study
FO/151-1/14	2014	Marine farm XXIII (MXXIII)	G4 (SS)	G2 (26.6) / G4 (23.5)	KX823934	Christiansen et al. 2017
FO/151-2/14	2014	Marine farm XXIII (MXXIII)	G4 (SS)	G2 (34.7) / G4 (26.6)	**OL631766**	Present study
FO/151-3/14	2014	Marine farm XXIII (MXXIII)	G4 (SS)	G2 (34.3) / G4 (27.4)	**OL631767**	Present study
FO/484-1/08	2008	Marine farm XXIV (MXXIV)	G4 (SS)	G2 (neg.) / G4 (27.3)	**OL631768**	Present study
FO/484-2/08	2008	Marine farm XXIV (MXXIV)	G4 (SS)	G2 (neg.) / G4 (28.0)	**OL631769**	Present study
FO/790-1/11	2011	Marine farm XXIV (MXXIV)	G2 (SS)	G2 (31.5) / G4 (neg.)	**OL631770**	Present study
FO/144-1/14	2014	Marine farm XXIV (MXXIV)	G4 (SS)	G2 (36.3) / G4 (29.9)	KX823933	Christiansen et al. 2017
FO/273-1/14	2014	Marine farm XXV (MXXV)	G4 (SS)	G2 (neg.) / G4 (28.2)	**OL631771**	Present study
FO/1085-1/14	2014	Marine farm XXV (MXXV)	G4 (SS)	G2 (neg.) / G4 (27.6)	**OL631772**	Present study

N/A: Not applicable.
Sequences from Virus ID in bold were included in the statistical analysis

Table S2: Ova and fry delivered to the six Faroese smolt farms (Smolt farms SI – SVI) from Faroese, Icelandic and Norwegian brood fish. Total number (millions) of eggs sold to the six smolt farms between 2008 and 2014

	Atlantic salmon smolt farms						
Year	SI	SII	SIII	SIV	SV	SVI	Total
2008	4.3	3.7	2.6	3.1	2.7	3.0	19.4
2009	3.3	1.7	2.8	3.1	2.8	3.7	17.4
2010	3.0	1.4	4.4	2.6	3.1	3.7	15.7
2011	6.9	1.4	2.2	2.3	3.9	4.1	17.4
2012	6.3	2.3	1.9	3.7	3.2	5.5	22.3
2013	4.7	1.8	1.7	3.4	3.6	5.3	21.5
2014	6.7	1.6	2.3	1.2	2.7	6.7	19.3
Total	35.3	13.8	18.0	19.2	22.0	32.1	140.0

Smolt farms SI, SV and SIV received ova from Icelandic, Norwegian and Faroese broodfish.
Smolt farm SIV received ova from Norwegian broodfish only from 2011 to 2014.
Smolt farm SIII received ova from Faroese broodfish only from 2008 to 2013.
Smolt farm SII had no hatchery and received fry directly from the Faroese broodfish farm and from smolt farm SI.

Table S3: Monthly ISAV-HPR0 Infection dynamics at the six ISAV smolt farms. Total number (n) of Atlantic salmon gills screened for ISAV and number (n) and percentage (%) tested ISAV positive by real-time RT-PCR throughout the study period from 2007 to 2014. Empty cells illustrate no sampling.

		Atlantic salmon freshwater smolt farms no.:																	
		SI			SII			SIII			SIV			SV			SVI		
		Total	ISAV pos.		Total	ISAV pos.		Total	ISAV pos.		Total	ISAV pos.		Total	ISAV pos.		Total	ISAV pos.	
Year	Month	n	n	%	n	n	%	n	n	%	n	n	%	n	n	%	n	n	%
2007	1																		
	2	80	0	0.0										80	0	0.0			
	3							80	0	0.0									
	4																		
	5							80	0	0.0							79	0	0.0
	6																		
	7																		
	8																		
	9																80	0	0.0
	10							80	0	0.0	80	0	0.0						
	11																		
	12																		
	Total	80	0	0.0				240	0	0.0	80	0	0.0	80	0	0.0	159	0	0.0
2008	1																		
	2																		
	3																		
	4	80	0	0.0	60	0	0.0	80	0	0.0	80	0	0.0	80	39	48.8	80	0	0.0
	5													40	0	0.0			
	6																		
	7																		
	8																		
	9																		
	10							48	0	0.0									
	11																		
	12							80	0	0.0	80	0	0.0				80	0	0.0
Total		80	0	0.0	60	0	0.0	208	0	0.0	160	0	0.0	120	39	32.5	160	0	0.0
2009	1	20	0	0.0	39	0	0.0	35	0	0.0				30	0	0.0			
	2	40	0	0.0							40	0	0.0	30	0	0.0	40	2	5.0
	3	40	0	0.0	40	0	0.0	35	0	0.0	40	0	0.0	30	0	0.0	40	40	100
	4	40	0	0.0	40	0	0.0	48	0	0.0	40	0	0.0	38	0	0.0	38	2	5.3
	5				40	0	0.0	40	0	0.0	40	0	0.0	35	0	0.0	40	0	0.0
	6	30	0	0.0	40	0	0.0	39	0	0.0	32	0	0.0	20	0	0.0	40	5	12.5
	7	30	0	0.0				40	0	0.0	29	0	0.0				30	9	30.0
	8	30	0	0.0										21	0	0.0			
	9	40	15	37.5				40	0	0.0	40	0	0.0	39	0	0.0	40	0	0.0
	10				40	0	0.0	40	0	0.0	40	0	0.0				40	0	0.0
	11	32	8	25.0							40	0	0.0	30	0	0.0	40	0	0.0
	12	30	2	6.7	30	0	0.0	40	0	0.0				34	0	0.0			
Total		332	25	7.5	269	0	0.0	357	0	0.0	341	0	0.0	307	0	0.0	348	58	16.7
2010	1	30	0	0.0	31	30	96.8	40	0	0.0	40	0	0.0	40	39	97.5	40	0	0.0
	2				30	30	100.0				40	0	0.0				29	0	0.0
	3	40	9	22.5				40	0	0.0	39	0	0.0	40	19	47.5	40	1	2.5
	4	40	0	0.0	40	0	0.0	40	0	0.0	40	0	0.0	43	0	0.0	40	3	7.5
	5																		
	6	40	0	0.0				45	0	0.0	40	0	0.0	36	5	13.9			
	7	40	0	0.0				40	0	0.0	40	0	0.0	40	23	60.0			
	8										40	0	0.0	40	22	55.0	31	0	0.0
	9	40	0	0.0	40	16	40.0							40	0	0.0			
	10	39	0	0.0	38	33	86.8	39	0	0.0	40	0	0.0	40	0	0.0	40	24	60.0
	11										40	0	0.0						
	12	40	0	0.0	40	33	82.5	40	0	0.0				40	0	0.0	40	1	2.5
Total		309	9	2.9	219	142	64.8	284	0	0.0	359	0	0.0	361	108	29.9	260	29	11.2
2011	1	40	0	0.0							40	0	0.0				40	0	0.0
	2	40	0	0.0	40	0	0.0	35	0	0.0	40	0	0.0	35	6	17.1	40	0	0.0
	3	40	0	0.0	40	0	0.0	40	0	0.0				40	0	0.0	40	0	0.0

	4	40	0	0.0	40	0	0.0	40	0	0.0	40	0	0.0	40	10	25.0			
	5	40	0	0.0										40	5	12.5	40	0	0.0
	6				40	0	0.0	25	0	0.0	40	0	0.0	40	5	12.5	40	22	55.0
	7	40	0	0.0	40	0	0.0	40	0	0.0	40	0	0.0	40	0	0.0	40	11	27.5
	8				36	0	0.0	40	0	0.0	39	0	0.0	40	14	35.0			
	9							40	0	0.0							40	1	2.5
	10	40	0	0.0	40	0	0.0	40	0	0.0	40	0	0.0	40	30	75.0	40	10	5.0
	11							40	0	0.0	40	0	0.0	40	0	0.0			
	12	40	0	0.0	40	0	0.0	40	0	0.0	40	0	0.0	40	7	17.5	39	10	25.6
Total		320	0	0.0	316	0	0.0	380	0	0.0	359	0	0.0	395	77	19.5	359	54	15.0
2012	1																		
	2																		
	3	40	0	0.0															
	4																		
	5													35	1	2.9			
	6				40	1	2.5										40	32	80.0
	7	40	0	0.0				40	0	0.0	40	0	0.0						
	8																		
	9																		
	10																		
	11													40	2	5.0			
	12				40	30	75.0										40	6	15.0
Total		80	0	0.0	80	31	38.8	40	0	0.0	40	0	0.0	75	3	4.0	80	38	47.5
2013	1																		
	2							40	0	0.0	40	0	0.0						
	3																		
	4	40	0	0.0															
	5																		
	6																		
	7													40	6	15.0	40	3	7.5
	8				40	6	15.0	40	0	0.0	40	0	0.0						
	9																		
	10																		
	11										40	0	0.0						
	12																		
Total		40	0	0.0	40	6	15.0	80	0	0.0	120	0	0.0	40	6	15.0	40	3	7.5
2014	1																		
	2																		
	3																		
	4				40	10	25.0	29	0	0.0	48	9	18.8				40	5	12.5
	5	34	0	0.0										40	0	0.0			
	6																		
	7																		
	8																		
	9																		
	10	40	2	5.0										44	0	0.0	40	23	57.5
	11				40	12	30.0	40	0	0.0	43	25	58.1						
	12										20	1	5.0						
Total		74	2	2.7	80	22	27.5	69	0	0.0	111	35	31.5	84	0	0.0	80	28	35.0

Table S4: **Yearly prevalence of the HPR0 subtypes G2 and G4 in the marine farms**. A total of 139 individual fish from the 75 fish cohorts in the 25 marine farms included in the present study were analysed by sanger sequencing and the G2 and G4 specific RT-qPCR assays

	HPR0 subtypes			
Year	G2 (%)	G4 (%)	G2 + G4 (%)	Total
2007	16 (36)	6 (13)	23 (51)	45
2008	4 (11)	17 (49)	14 (40)	35
2009	1 (7)	9 (60)	5 (33)	15
2010	2 (22)	2 (22)	5 (56)	9
2011	1 (25)	0 (0)	3 (75)	4
2012	2 (25)	5 (63)	1 (12)	8
2013	0 (0)	4 (100)	0 (0)	4
2014	0 (0)	6 (32)	13 (68)	19
Total	26 (19)	49 (35)	64 (46)	139

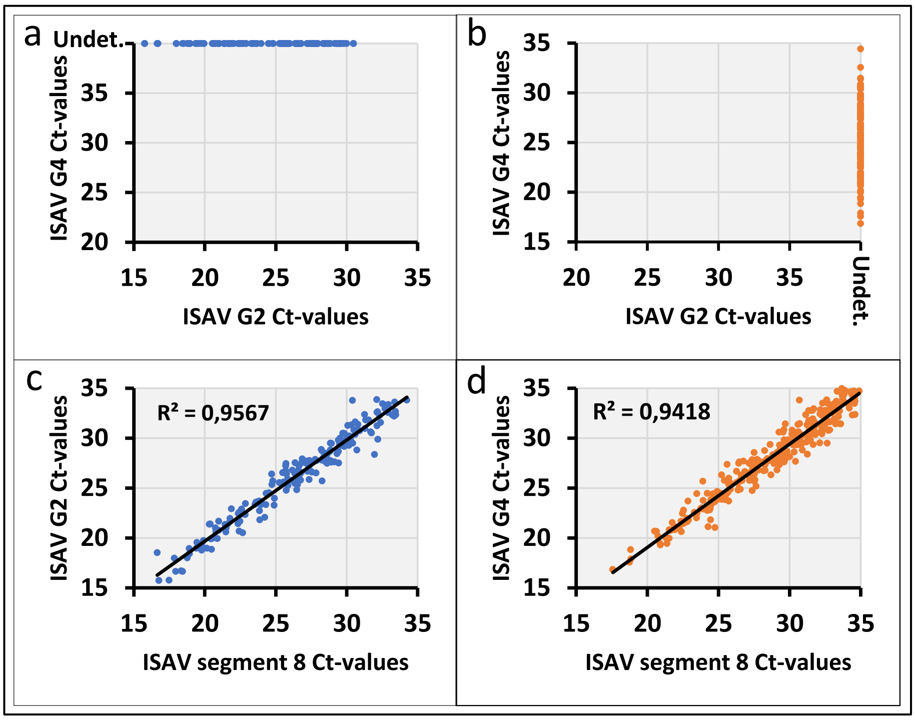

Figure S1: Scatter plot demonstrating the specificity and sensitivity of the G2- and G4-specific real-time RT-PCR assays. The G2 and G4 assays demonstrated 100% specificity (a and b) and a high sensitivity (c and d) comparable to the duplex ISAV real-time RT-PCR segment 8 assay (Christiansen *et al*. 2011, 2017) over a wide dynamic range of viral load (Ct-values ranging from 15 to 35). In a 149 HPR0 positive broodfish were tested positive with the G2 assay and negative with the G4 assay (G4 negative Ct-values were set to 40 for visual presentation). In b 214 HPR0 positive smolts were tested positive with the G4 assay and negative with the G2 assay (G2 negative Ct-values were set to 40 for visual presentation). The Scatter plot demonstrated high correlation (R2 > 0.94) between the Ct- values of ISAV segment 8 versus G2 (191 broodfish) (c) and ISAV segment 8 versus G4 (331 smolt) (d).

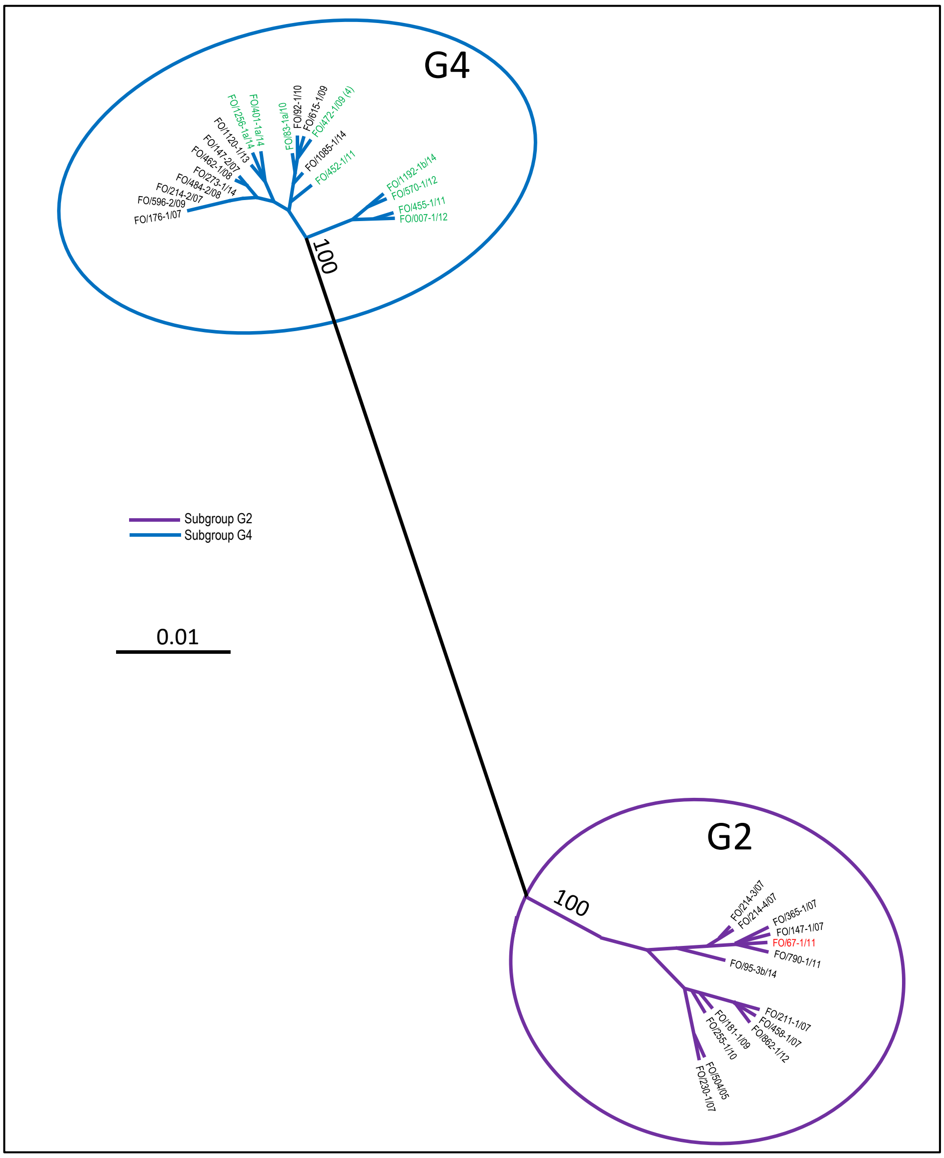

***Figure S2**: Phylogenetic radial tree showing the relationship between all 34 unique HPR0 variants clustering in G2 (14 variants) or G4 (20 variants) from Faroese (FO) broodfish (red variant ID), Faroese smolt (green variant ID) and Faroese marine salmon (black variant ID) (for further details, see Table S1). The phylogenetic analysis was performed on 1154 nucleotides (nt 61 to 1214 relative to the start codon) of the haemagglutinin esterase (HE) gene. Branch length reflects genetic distance. Bar, 0.01 substitutions per nucleotide side*

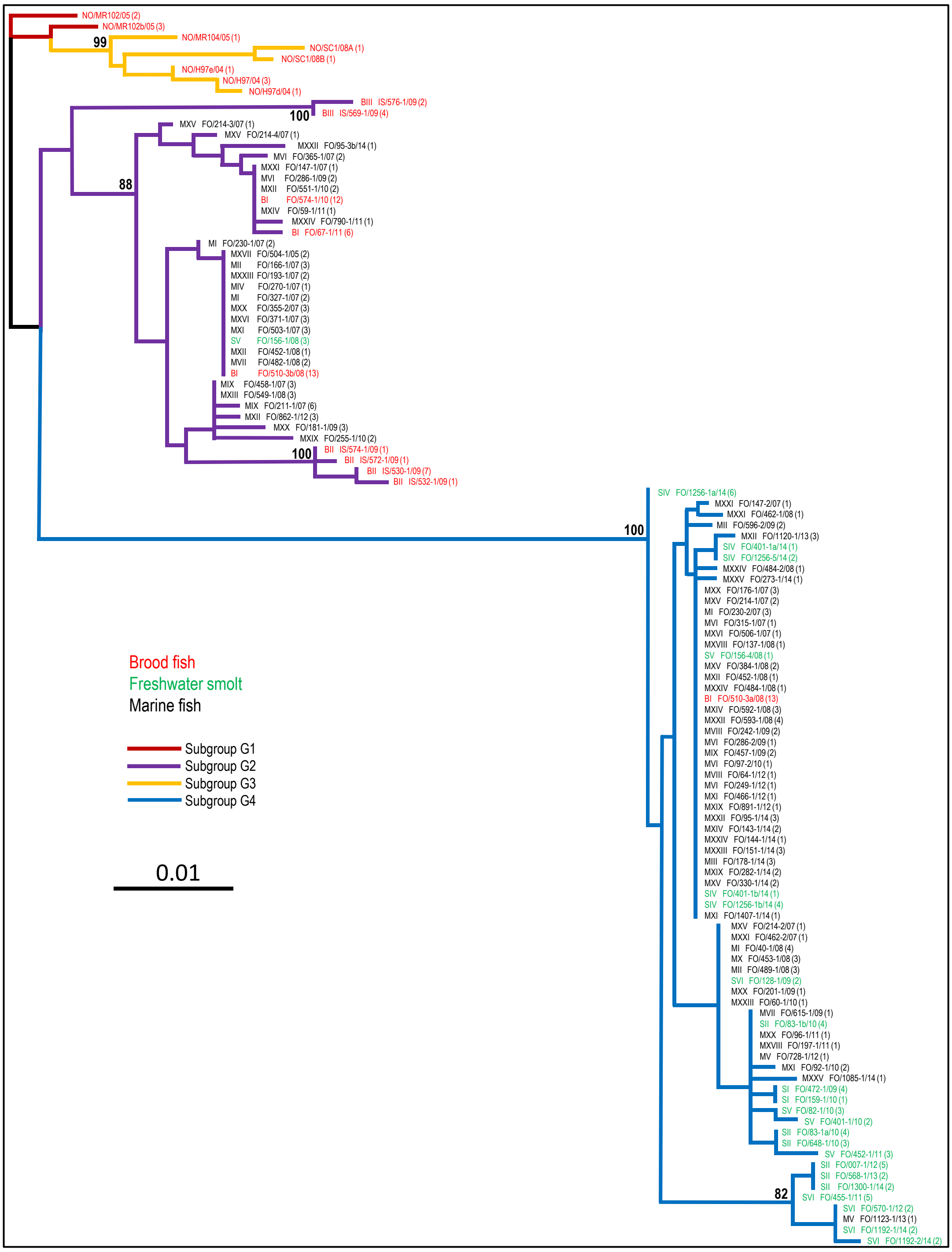

Figure S3: *A Maximum Likelihood phylogenetic three showing the relationship between HPR0 variants from the three production stages in the Faroe Islands detected between 2005 and 2014, as well as Norwegian and Icelandic Broodfish. The 35 Faroese HPR0 variants (see Figure S2) were identified among a total of 99 HPR0 positive fish cohorts (i.e. 3 Broodfish, 21 smolt and 75 marine fish cohorts) included in the present study. Included in the phylogenetic three is representative HPR0 HE-sequences from all Norwegian (NO) broodfish, Icelandic (IS) broodfish (BII and BIII) and Faroese (FO) broodfish (BI), smolt (SI – SVI) and marine farming sites (MI – MXXV). The 99 representative FO HPR0 variants were all collected at different timepoints and in different fish cohorts. Number in brackets represents identical HPR0 variants from different fish from the same fish cohort. The phylogenetic analysis was performed on 1154 nucleotides (nt 61 to 1214 relative to the start codon) of the haemagglutinin esterase (HE) gene. Branch length reflects genetic distance. Bar, 0.01 substitutions per nucleotide side.*
